# Enzymatic Activity and Physicochemical Properties of Soil Profiles of Luvisols

**DOI:** 10.3390/ma14216364

**Published:** 2021-10-24

**Authors:** Anna Piotrowska-Długosz, Mirosław Kobierski, Jacek Długosz

**Affiliations:** Department of Biogeochemistry and Soil Science, Laboratory of Soil Science and Biochemistry, Faculty of Agriculture and Biotechnology, Bydgoszcz University of Science and Technology, Bernardyńska 6 St., 85-029 Bydgoszcz, Poland; kobierski@pbs.edu.pl (M.K.); jacekd@pbs.edu.pl (J.D.)

**Keywords:** depth pattern, lessivage, Luvisols, physicochemical properties, soil enzymes, soil profile

## Abstract

Most studies on soil enzymes are focused on the upper horizons of the soil profile, even though they transform the soil organic matter at every depth of the soil profile. The aim of this work was to investigate the distribution of β-glucosidase (GLU), nitrate reductase (NR), urease (UR), phosphatase (PHA), dehydrogenase (DHA) and catalase (CAT) activity through 14 trunked soil profiles of the Luvisols formed from a glacial till. The content of microbial biomass carbon (MBC) as well as physicochemical properties such as organic carbon (C_ORG_), total nitrogen (N_TOT_), available P, K and Mg, soil density and porosity, pH in KCl and fractional composition were also studied. In general, enzymatic activity was highest in the top 30 cm layer of the profiles and decreased progressively towards the deeper horizons. The exceptions were the NR activity, which was active only in the Ap horizon and whose activity decreased sharply to nearly zero in the Bt horizon and parent rock, and the PHA activity, which was highly active even in the parent rock depth. The decreased availability of carbon and nutrients was the main driver of decreases in microbial abundance and enzymatic activity with depth. The enzymatic activity, when expressed on a C_ORG_ and MBC basis, behaves differently compared to the activity expressed on a soil mass basis. The activity decreased (NR), increased (PHA, UR), showed no clear pattern (GLU) or the changes were not significant (DHA, CAT). The content of C_ORG_, N_TOT_, K and P_AVAIL_ generally decreased with depth, while for Mg, there was no clear direction in the profile distribution. Future studies to characterize the substrate distribution within the soil profile and enzyme stability will provide further insight into the controls on nutrient cycling and related enzymes throughout the soil profiles.

## 1. Introduction

Soil enzymes mediate decomposition of soil organic matter and catalyze the main processes in carbon, nitrogen and phosphorus transformations [1,2]. Measuring the enzymatic activity in soils has a long tradition in the evaluation of changes in the soil quality that are related to different natural and human-induced factors, especially agricultural practices [3,4]. Each type of soil has its own characteristic pattern of specific enzymes and its own inherent level of enzymatic activity, which depends on its origin and development conditions and on the content of organic matter [5]. During the last few decades, much effort has been devoted to investigating the soil enzymes in the surface soil layers, where the enzyme activity is expected to be higher than in the lower layers of the profile due to a higher activity of soil microorganisms and root system density, which are the main sources of the enzymes in soil [6]. Less is known, however, about their activity in the deeper horizons of the profile, despite the fact that the subsurface enzymes play an important role in soil formation, ecosystem biogeochemistry, contaminant degradation and, consequently, the maintenance of groundwater quality [2,4,7,8].

Although some attempts have been made to determine the patterns of the distribution of the soil enzyme activity in various soil profiles, the relative importance of the factors that influence the level of their activity through a specific profile has not yet been clearly established [6,9]. The enzymatic properties throughout the soil profile depend mainly on the concentration and quality of the carbon substrates within the soil profile. These, in turn, are determined by the quantity and quality of plant residues and root exudates, which are the sources of energy and nutrients for soil microorganisms as well as the source of the substrates for the soil extracellular enzymes. Although the highest resources of the available C substrates are found in the topsoil (up to 30 cm), large amounts of organic C (about 60%) are stored in the deeper soil horizons. That is why, due to their high potential to store carbon, the subsurface horizons need to be considered in the global C sequestration [10]. Carbon in the deep soil horizons is more stable and has a long turnover time compared to the C in the surface horizons [11]. In the subsoil horizons, where fresh C inputs are smaller compared to the surface soil horizons and where a large proportion of soil C is stabilized via its interaction with the mineral surfaces, only a small amount of organic carbon is easily accessible for microorganisms [12]. This is reflected in the microbial biomass and enzymatic activity, which typically decrease with soil depth. The vertical distribution of enzymatic activity, similar to the horizontal distribution, is also affected, among others, by the soil pH, soil texture, soil nutrient content and air–water conditions [2,6,7,8,13].

Luvisols represent averagely fertile soils, which are quite rich in nutrients for plants. Considering the large area that they cover, they constitute the basis for agricultural production in Poland [14]. The soils selected for the study dominate in a young morainic area of northern Poland because of the climatic conditions of the area, with the advantage of rainfall over evaporation. Luvisols of the studied sites were morphologically changed as a result of the erosion that was caused by human-induced factors, mainly related to agricultural practices [15,16]. This mainly concerns agricultural landscapes where the erosion processes caused translocation of soil mass from convexities and from the upper areas of the cultivated hillslopes to concavities and the lower areas of the field, where the original soil profile becomes buried. The truncation of the A and E horizons is the result of this erosion [15,16,17,18]. The rate of the truncation depends on the granulometric composition and the permeability of the parent material of the soil and climate conditions. All this results in the formation of the Ap horizon from either the E horizon or from the upper part of the Bt level, and therefore, these soils are morphologically similar to Cambisols. The presence of the argic horizon is evidence that the soils are Luvisols. In fact, a recent micromorphological analysis of these soils showed the occurrence of numerous oriented clay bodies in thin sections of the Bt horizon. It was also found that the ratio of fine clay to total clay in the argic horizon is greater by ≥1.2 times than the ratio in the overlying coarser textured horizon [19]. Additionally, the confirmation of the occurrence of the argic horizon is the presence of clay coating linings as well as clay coatings covering the vertical and horizontal surfaces of soil aggregates in the Bt horizon [20]. Besides the morphological changes within the soil profile, the truncation process can modify a wide range of physicochemical features (e.g., infiltration rate) [19]. Additionally, this process can modify the enzymatic activity down the soil profile as a result of changes in bulk density and porosity, which in turn affects the penetration depth of plant roots or microbial diversity, which are the main sources of enzymes in soil. The intensity of the truncation process as well as the transformation rate of these soils are affected by the differentiation of the parent material, genesis and the age of morainic plateau, the relief, and the sustainability and intensity of human agricultural activity [15,16,20].

To assess whether the long-term lessivage and truncation processes influence the enzymatic activity of the transformed soils in the same way, regardless of their location on different moraines, the soil profiles used for this research were located in three different lake districts (Krajna, Chodzież and Chełmno Lakelands), which had been formed during two phases of the last glaciation that occurred in northern Poland.

The main aim of the study was to investigate the changes in the potential and specific enzyme activity of the C, N and P cycles (β-glucosidase, nitroreductase, urease, phosphatase) as well as the activity of dehydrogenase and catalase at increasing soil depths in 14 profiles of Luvisols subjected to soil transformation connected with the lessivage process across three mesoregions of the South Baltic Lake District (Krajna Lakeland, Chodzież Lakeland and Chełmno Lakeland). We determined a set of soil enzyme activities that are most important in determination of the fertility of arable soils and are responsible for basic nutrient transformation (C, N and P). Additionally, soil dehydrogenases and catalase were determined as the general indicators of soil biological activity. We hypothesized that soils that had been subjected to a long-term lessivage process would have modified physicochemical properties across the horizons to different degrees, which also caused differences in their microbial and enzymatic properties. We hypothesized that the availability of C and nutrients (e.g., K and P) would be a major driver of the vertical distribution of soil enzymatic activity, wherein the total enzyme activity would decrease with depth, similar to the decrease in nutrient availability and microbial biomass content. We also hypothesized that a specific enzymatic activity (i.e., activity per unit of organic carbon and microbial biomass C) would increase with depth, thereby reflecting a greater microbial potential to produce enzymes in response to decreased availability of carbon and nutrients. Our further objective was to investigate the influence of cultivated plants (winter wheat and winter rape) on the enzymatic distribution down the soil profile. We predicted that the influence of plants would be the highest in the surface horizon, would decrease with depth, and would mainly be affected by the parent material in the subsoils.

## 2. Materials and Methods

### 2.1. Study Sites and Sampling

The study was conducted on 14 soil profiles of Luvisols [19], developed from loamy ground morainic deposits of the Vistulian glaciation, which are located in three mesoregions of the South Baltic Lake District, e.g., the Krajna Lakeland (5 profiles), the Chodzież Lakeland (4 profiles) and the Chełmno Lakeland (5 profiles) (Cuiavia-Pomerania Province, central Poland). All of the studied soils are located in areas that have a long-term history of agricultural use and were formed from the same glacial till, deposited during the Vistulian glaciation. The regions differed in the age of the deposition of the parent material and the time at which the soil formation factors were affected (between 20 ka BP—Chodzież Lakeland to 16.5 thousand years—Chełmno Lakeland). A long-term lessivage process, which occurred in the studied soils, modified their properties with varying degrees. This was confirmed by the differentiation in the thickness of the Bt horizon (39–78 cm), which consists of two parts (Bt1 and Bt2) in all of the soil profiles. The first sub-horizon (Bt1) was about 19–33 cm thick (average for the regions) but had a higher clay content. The second sub-horizon (Bt2) had an greater thickness (20–45 cm) but a lower clay content. In all of the profiles studied, the effects of the truncation process was found, which resulted in the lack of the eluvial horizon (E), which was absorbed by the Ap horizon [15,16]. This resulted in a lower content of the clay fraction in the surface horizon than in the parent material. In the studied profiles, the following thicknesses of the horizons were found: Ap horizons 25–32 cm, the Bt1 19–33 cm, the Bt2 20–45 cm, the Ck1 20–60 cm and the Ck2 15–35 cm. The climate in these regions is temperate with well-below-zero (°C) temperatures in the winter, an average annual temperature of 7 °C and average precipitation of 550 mm year^−1^. 

The soil samples were collected from the fields with winter rape immediately after the harvest (August) in order to avoid any direct fertilization or vegetation effects. The resulting outcrop was 2 m × 2 m, down to a depth of 1.5 m. The soil samples were taken by gently scratching each soil pit wall for each horizon. Three soil samples from the middle portion of each horizon were collected with a gouge auger for stepwise sampling, pooled and thoroughly mixed. The soil samples from the 0–30 cm layers were placed in plastic containers (which permitted gas exchange) and chilled to 4 °C in order to minimize any changes in the populations of microorganisms. The samples taken from the deeper layers of the soil were placed in sealed containers, which generated an atmosphere with a reduced oxygen content, and chilled to 4 °C. The enzymatic activity was determined within three weeks. The soil samples used to determine the selected physicochemical properties were air-dried and sieved (2 mm).

### 2.2. Physicochemical Properties

The particle size was defined using the Cassagrande method, as modified by Prószyński, and the sand fraction content was determined using the sieving method [21]; the pH of a solution of 1 M KCl was measured using the potentiometric method in 1:2.5 soil:solution suspensions [22]; the content of total organic carbon (C_ORG_) and total nitrogen (N_TOT_) was determined using a dry combustion CN analyzer (Vario Max CN, Elementar Analysensysteme GmbH, Hanau, Germany),. The content of available magnesium (Mg) and potassium (K) was determined using atomic absorption spectrometry (AAS) (PU 9100X, Philips, Cambridge, Great Britain) after extraction with 0.0125 M CaCl_2_ (Mg) and the Egner-Riehm DL method (available P and K) [23]. The available phosphorus (P) was assayed using the vanadium-molybdenum method [24]. The bulk density was determined with the method of applying volume cylinders (100 cm^3^) [19]. A metal cylinder was pressed into the soil, and the moist sample mass was recorded and weighed. The sample was then oven-dried (105 °C) and weighed again. The bulk density was recorded as the ratio of dry mass to volume at the determined water content. Measurements of each sample were made in triplicate, and the mean value was calculated. The soil porosity was defined based on the specific density (data not presented) and the bulk density [25]. The analyses of the physical and chemical properties were analyzed in triplicate.

### 2.3. Enzymatic Activity

The enzyme activity was determined in fresh, moist and sieved (<2 mm) soil. The soil dehydrogenase activity was determined according to Thalmann [26] by using triphenyltetrazolium chloride as an electron acceptor and a Tris-HCl buffer at pH 7.6. Nitrate reductase activity (NR) was measured according to Kandeler [27] using KNO_3_ as the substrate. After incubating the soil samples at 25 °C for 24 h and the controls at −20 °C, the released nitrates were extracted using a 4 M KCl solution and then determined colorimetrically at 520 nm. Catalase activity (CAT) was assayed according to Johnson and Temple [28]. A mixture of soil, distilled water and 0.3% hydrogen peroxide solution was shaken for 20 min and then H_2_SO_4_ was added to stop the reaction. After filtration, the residual H_2_O_2_ was determined by titration with KMnO_4_. To eliminate any possible overestimation of the enzyme activity due to the chemical reduction of the H_2_O_2_ that was added, a correction was made for the autoclaved soil (0.1 MPa, 120 °C, 30 min). The soil phosphatase (EC 3.1.3.2) and β-glucosidase (EC 3.2.1.21) activity was determined using p-nitrophenyl phosphate (0.115 M) and p-nitrophenyl-β-D-glucopyranoside (0.05 M) as the substrates, respectively. Specific enzyme assay procedures (buffers, temperature and duration of incubation and reaction stop) were used as reported in Tabatabai and Bremner [29] and Eivazi and Tabatabai [30]. The concentration of p-nitrophenol was determined at 400 nm after the addition of NaOH and CaCl_2_ for the phosphatase and a Tris/NaOH buffer (pH 10.0) and CaCl_2_ for the β-glucosidase. The same procedures were followed for the controls as those for the soil enzyme assays, with the only difference being that the substrates were added to the reaction mixture after incubation and immediately prior to stopping the reaction. Soil urease activity was determined according to Kandeler and Gerber [31] by monitoring the release of ammonium from the soil treated with urea as a substrate and incubated with a borate buffer at pH 10.0. The activity of all the enzymes was calculated using the standard curves. 

One unit of enzyme activity was defined as the amount of product released by 1 g of dried soil per 1 min (for CAT) and 1 h (for DHA, NR, PHO, GLU and UR). 

### 2.4. Microbial Biomass Carbon Content

A fumigation-extraction method was used to estimate microbial biomass C (MBC), with extractable C converted to microbial C using a standard factor (Kc = 0.38) [32]. A soil sample was placed in a desiccator with wet tissue paper and a beaker with 25 mL of chloroform with a few boiling chips. The desiccator was evacuated until the chloroform boiled vigorously and was placed in the dark at 25 °C for 24 h. After incubation, the chloroform was removed by repeated evacuation. Both fumigated and unfumigated soil samples were then extracted with 0.5 M K_2_SO_4_ for 30 min and analyzed for soluble C, as proposed by Vance et al. [32]. The MBC/C_ORG_ (%) ratio was also calculated [33].

### 2.5. Statistical Analysis

The data set was evaluated using classical statistics, and the mean, range, standard deviation and coefficient of variation (CV), which is the ratio of the standard deviation (SD) to the mean value times 100, were calculated. The studied properties did not show a normal distribution and, therefore, were transformed accordingly (Gaussian anamorphosis transformation). Since the transformation improved the normality of the properties, further analyses were performed with the corrected data. A two-way analysis of variance (ANOVA) was performed to determine the significance of the regions being studied (Krajna Lakeland, Chodzież Lakeland and Chełmno Lakeland) and the properties being studied at the same genetic horizon as well as to determine the effect of soil depth (five horizons up to 150 m) on these properties. Additionally, a one-way analysis of variance was performed to assess the significance of the influence of cultivated plants (winter rape and winter wheat) on the properties being studied. Any significant differences between the means were determined using the Tukey’s Post Hoc Test with 95% confidence interval. A classification scheme was used to identify the extent of the variability in the soil properties based on their CV (%) values, which was calculated as the ratio of the standard deviation (SD) to the mean value times 100. The ranges of 0–15%, 16–35%, and >36% indicate little, moderate and high variability, respectively [34]. An analysis of correlation was performed to investigate the relationship between the studied properties, taking into account all of the data (all of the horizons down the soil profile were considered together) from each study site separately. The correlation matrix of the properties was based on Pearson’s correlation coefficients, using *, ** and *** to indicate the 95%, 99% and 99.9% probability levels, respectively. Statistical analyses were carried out using Statistica 8.1 for Windows.

## 3. Results

### 3.1. Physicochemical Properties and Microbial Biomass Carbon (MBC)

**Figure 1:**
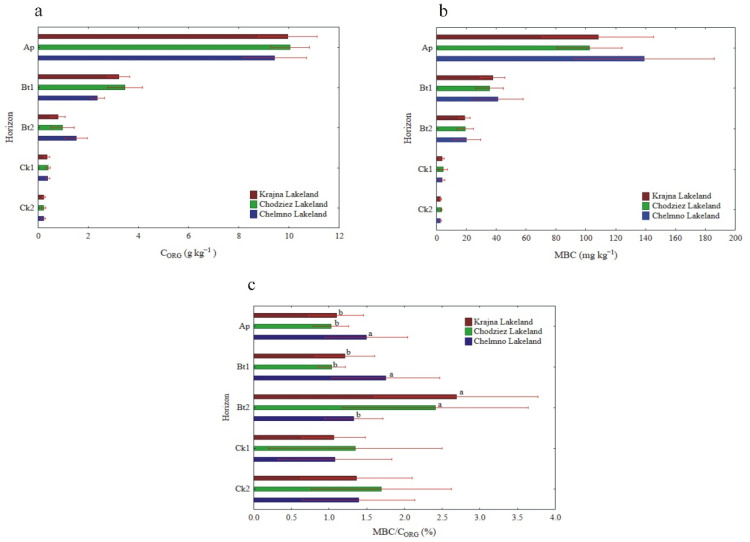
The content of C_ORG_ (**a**), MBC (**b**) and MBC/C_ORG_ ratio (**c**) at different soil depths (mean and SD). Different lowercase letters indicate significant differences (*p* < 0.05) between study sites (within the same genetic horizons of the profile). C_ORG_—organic carbon; MBC—microbial biomass content.

The soils showed a reaction (pH in KCl) from slightly acid to neutral, whereas in the parent material, which was rich with CaCO_3_ (data not presented), the reaction was neutral and alkaline (Table 1). The values of hydrolytic acidity (Hh) showed the opposite behavior compared to pH in KCl and were the highest in the Ap-Bt2 horizons and significantly lower in the parent material. The C_ORG_, MBC and N_TOT_ content was the highest in the Ap horizon and decreased significantly across the soil horizons (Table 1, Figure 1a,b). The C/N ratio at the surface level was typical for soil with an adequate microbiological activity and was similar to the optimal ratio of 10:1. The values of the C/N ratio also decreased with the depth of the soil profiles (Table 1). The ratio of MBC/C_ORG_ ranged between 1.06 and 2.78% and was significantly differentiated between the studied regions (in Ap, Bt1 and Bt2 horizons). A significantly higher ratio in the Bt2 layer than in the Ap and Bt1 horizons was observed in the Chełmno Lakeland, while in the Bt2 horizon, the tendency was opposite (Figure 1c). For all of the studied profiles, the highest MBC/C_ORG_ ratio was observed in the Bt2 horizon, while in the other layers, the ratio was lower but not statistically significant (Table 1). With regard to the study regions, there was a significantly higher MBC/C_ORG_ ratio in the Ap and Bt1 horizons in the Krajna Lakeland compared to the Chodzież and Chełmno Lakelands, while in the Bt2 horizon, the situation was the opposite. In the two deepest horizons, the ratio was not differentiated, regardless of the study region (Figure 1c).

**Figure 2:**
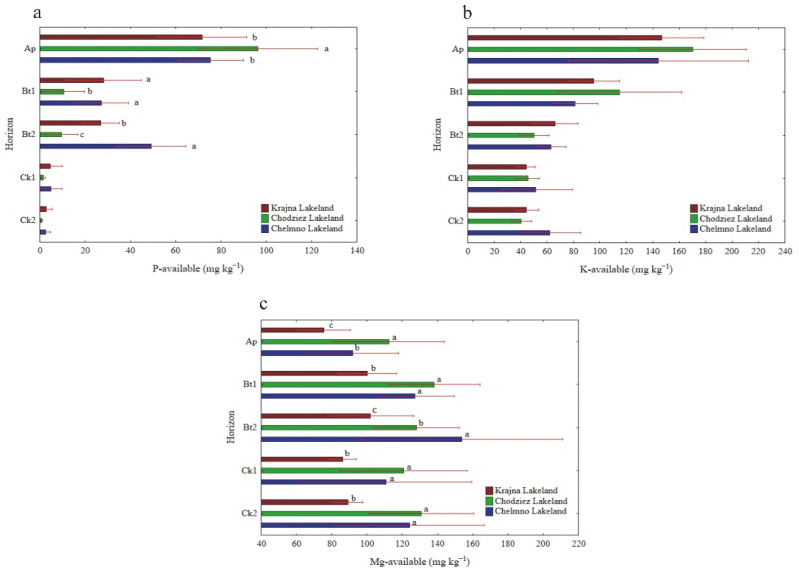
The content of available P (**a**), K (**b**) and Mg (**c**) at different soil depths (mean and SD). Different lowercase letters indicate significant differences (*p* < 0.05) between study sites (within the same genetic horizons of the profile).

**Figure 3:**
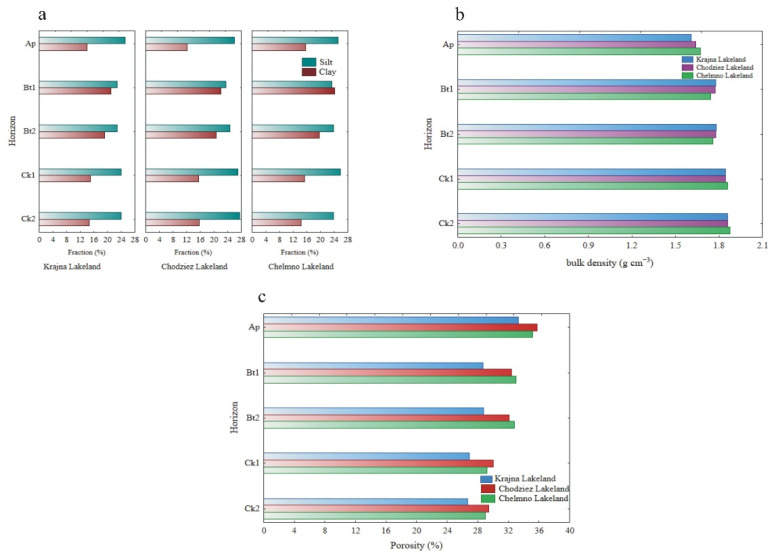
The content of silt and clay (**a**) bulk density (**b**) and porosity (**c**) at different soil horizons.

The available P and K content was differentiated among soil horizons, and the concentration decreased significantly from the Ap horizon (80.4 and 152.8 mg kg^−1^, respectively) to the parent material (Table 1, Figure 2a,b). Additionally, the P_AVAIL_ content was region specific; there was a significantly higher content of this element in the Ap horizon in the Chodzież Lakeland compared to the Chełmno and Krajna Lakelands. In the Bt1 and Bt2 horizons, an inverse relationship was observed (Figure 2a). In turn, there were no significant changes in the Ck1 and Ck2 horizons among the studied regions (Figure 3b). The K_AVAIL_ content was not significantly differentiated among the studied regions. In contrast to the distribution of the P and K, there was no clear direction in the Mg distribution in the soil profiles (Table 1). The subsurface horizons (Bt1 and Bt2) were generally richer in Mg (120.7–127.9 mg kg^−1^) than the Ap horizon (92.9 mg kg^−1^). A significantly higher content of Mg was found in the soil profiles that had been collected from the Chodzież Lakeland compared to the Chełmno and Krajna Lakelands, except for the Bt2 horizon (Figure 2c). 

Neither the silt nor the clay content (%) was significantly differentiated regardless of the study region (Figure 3a). In turn, when the clay content was considered to be independent of the study regions, it was the highest in the Bt1 horizon (22.4%), followed by the Bt2, Ck1, Ck2 and Ap horizons (Table 1).

The silt content was not significantly differentiated across the soil horizons. The bulk density ranged from 1.64 to 1.87 g cm^−3^ (Table 1), which is typical for mineral soil. Both bulk density and porosity values were not statistically significant, regardless of the study region (Figure 3b,c). However, an inverse relationship was found for these properties when the depths of the soil profiles (mean for 14 profiles) were considered. The bulk density increased slightly with the depth and reached the highest values in the parent material, while the porosity had the highest values in the Ap horizon (35.9%) and decreased down the soil profiles (Table 1, Figure 3b,c).

### 3.2. Soil Enzymatic Activity

#### 3.2.1. Enzymatic Activity Expressed Per Soil Unit

The enzymatic activity expressed per soil unit was the highest in the surface horizons of all of the profiles (Table 2, Table 3 and Table 4). Five out of six of the enzymes that were studied had most of their enzymatic activity within the first 0–25 (0–32) cm, which sharply decreased with increasing depth. In turn, the phosphatase activity had a progressively decreasing pattern toward the lower horizons and even had marked activity below a depth of 1 m.

All of the enzymes had a significant variability in the individual horizons, which was confirmed by the high standard deviation (SD) and CV values (Table 2, Table 3 and Table 4).

Both oxidoreductases were the most active in the topsoil and decreased towards the deeper soil horizons in all of the study regions. Soil DHA and CAT in Ap horizons reached only 2.1–3.6% and 6.2–8.4% of the activity noted in the C horizons (Table 2). In all profiles studied, the UR activity was the highest in the Ap horizons (2.68 to 3.76 mg N-NH_4_^+^ kg^−1^ soil h^−1^), followed by the deeper layers, for which the activity ranged between 1.06 and 0.25 mg N-NH_4_^+^ kg^−1^ soil h^−1^. In the soil profiles of the Krajna and Chodzież Lakelands, there were no significant differences in the UR activity between the Bt2, Ck1 and Ck1 horizons, while in the profiles from the Dobrzyń Lakeland, there were no significant differences in the activity of this enzyme in any of the sub-surface horizons (Table 3). Almost all (about 95%) of the NR activity was located in the surface horizons, within a range of 0.88–1.98 mg N-NO_2_ kg^−1^ soil h^−1^ (Table 3). Below the Ap horizon, the NR activity decreased sharply, down to less than 0.1 mg N-NO_2_ kg^−1^ soil h^−1^ in the Bt horizon and parent material. Among the studied enzymes, the NR activity had the greatest variability down the profile, which was confirmed by CV values, which ranged from 16.9 to 137.9%. Although the PHA activity (Table 4) was the highest in the Ap horizon, its activity decreased down the profile to the lowest degree compared to the other enzymes and had marked activity even in the parent material. At the surface, the PHA activity ranged from 63.7 to 67.0 mg *p*NP kg^−1^ soil h^−1^. An almost 50% decrease in the PHA activity was observed in the Bt1 horizon compared to the surface horizon. Over 80% of the GLU activity was concentrated in the upper horizons of the profiles and ranged from 43.3 to 51.0 mg *p*NP kg^−1^ soil h^−1^ (mean for all of the profiles). The enzyme activity decreased to a value of 4.43 mg *p*NP kg^−1^ soil h^−1^ in the Bt1 layer, while in the parent material, the activity level was lower than 1 mg *p*NP kg^−1^ soil h^−1^ in the Krajna and Chodzież Lake Districts (Table 4). 

There was no clear pattern in the variation of enzyme activities in the soil profiles across the study regions (Table 2, Table 3 and Table 4). There was a significantly higher DHA activity in the 

Ap horizon in the soil profiles from the Chełmno Lakeland than in the Krajna and Chodzież Lakelands, while in both B horizons, the opposite pattern was found. 

At the deepest horizon, the study regions had no impact on the DHA activity (Table 2). The study regions did not differentiate the CAT activity in the Ap horizon, while in the Bt1-Ck1 layers, the activity was significantly higher in the Krajna and Chodziez Lakelands than in the Chełmno Lakeland. In the Ck2 horizon, there was an opposite pattern of this activity (Table 2). A higher UR activity was found in the Chodzież region than in two other regions, but it was true across the Bt2-Ck2 horizons. The NR activity was not significantly differentiated in the Bt2-Ck2 horizons among the regions, while in the upper layers, the activity was higher in the Krajna and Chełmno Lakelands than in the Chodzież region (Table 3). The study regions did not significantly affect the PHA activity, but only in the Ap and both B horizons. Below, there was a significantly higher PHA activity in the profiles that had been in the Chodzież region than in the other two regions (Table 4). 

**Figure 4:**
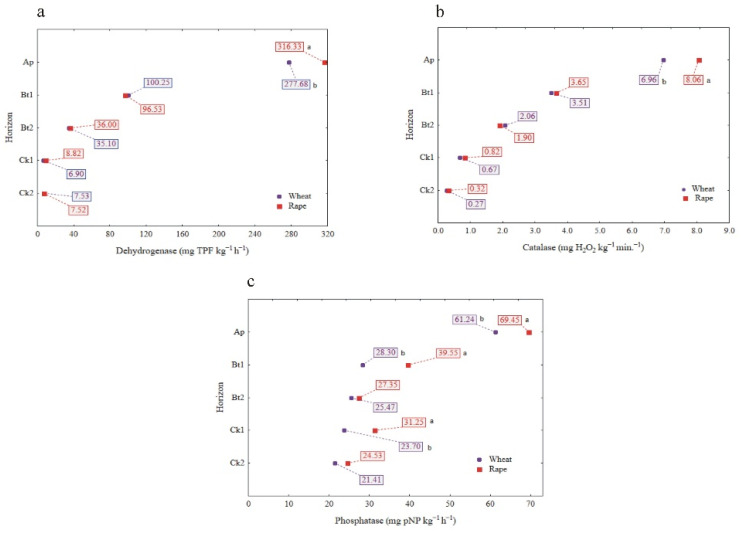
The influence of cultivated plants (winter wheat and winter rape) on enzyme activity: DHA(**a**), CAT (**b**) and PHA (**c**). Different lowercase letters indicate significant differences (*p* < 0.05) between cultivated plants in the same genetic horizons of profiles (mean values for the study sites).

The influence of cultivated plants (winter wheat and rape) on the enzymatic distribution down the profiles is presented in Figure 4a–c. Both the CAT and DHA activity in the Ap horizons of the soil profiles (means for considered regions) were slightly (but statistically significant) higher in the soil samples taken under winter rape cultivation than those for winter wheat cultivation. Below the Ap horizon, there were no significant differences in the activity of CAT and DHA as affected by the cultivated plants (Figure 4a,b). In turn, the PHA activity was significantly higher in the soil profiles selected from the fields under winter rape cultivation as compared to winter wheat cultivation, and this was true for the Ap, Bt1 and Ck1 horizons (Figure 4c).

#### 3.2.2. Enzymatic Activity Expressed on an Organic Carbon (C_ORG_) and Microbial Biomass Carbon (MBC) Basis

**Figure 5:**
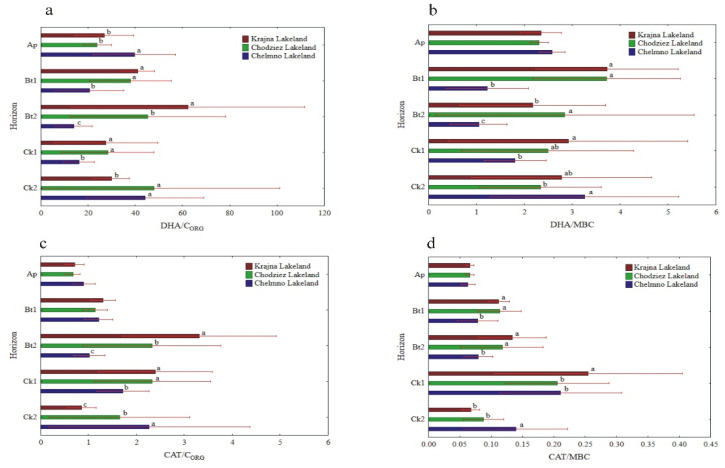
Activity of soil DHA and CAT at different soil horizons, expressed on an organic carbon (C_ORG_) and microbial biomass carbon (MBC) basis; DHA/C_ORG_ (**a**), DHA/MBC (**b**), CAT/C_ORG_ (**c**) and CAT/MBC (**d**). Different lowercase letters indicate significant differences (*p* < 0.05) between the study sites (within the same genetic horizon).

**Figure 6:**
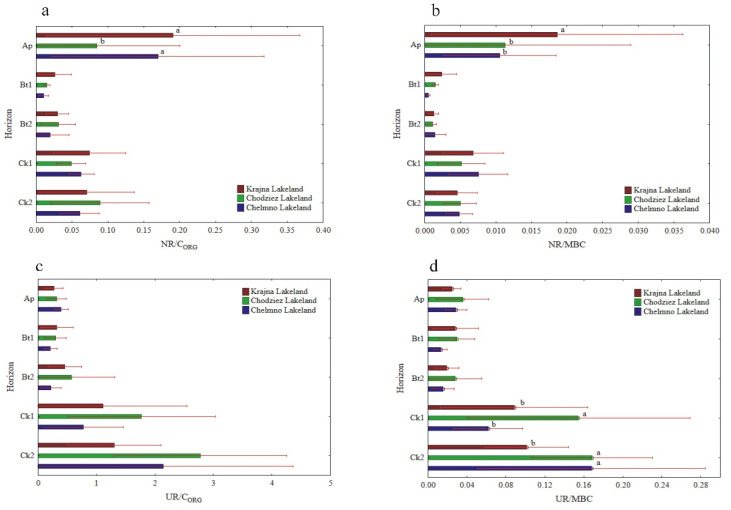
Activity of soil NR and UR at different soil horizons, expressed on an organic carbon (C_ORG_) and microbial biomass carbon (MBC) basis; NR/C_ORG_ (**a**), NR/MBC (**b**), UR/C_ORG_ (**c**) and UR/MBC (**d**). Different lowercase letters indicate significant differences (*p* < 0.05) between the study sites (within the same genetic horizon).

**Figure 7:**
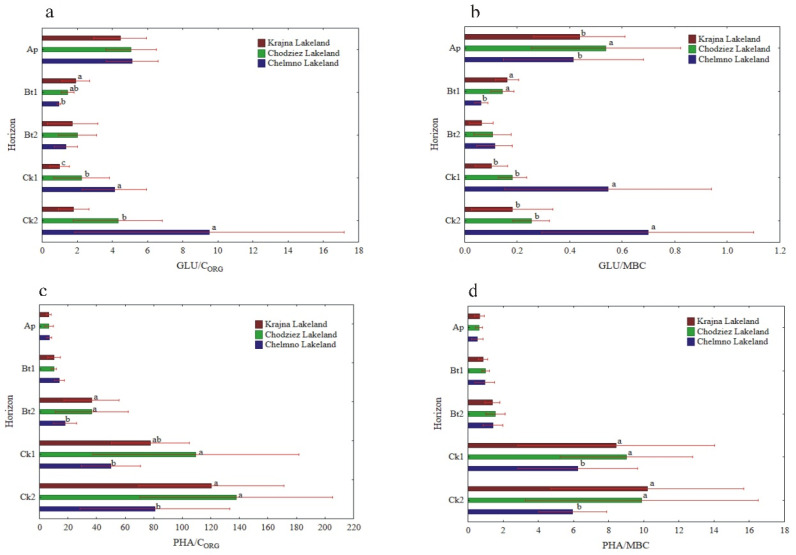
Activity of soil GLU and PHA at different soil horizons, expressed on an organic carbon (C_ORG_) and microbial biomass carbon (MBC) basis; GLU/C_ORG_ (**a**), GLU/MBC (**b**), PHA/C_ORG_ (**c**) and PHA/MBC (**d**). Different lowercase letters indicate significant differences (*p* < 0.05) between the study sites (within the same genetic horizon).

The specific enzymatic activity (expressed on C_ORG_ and MBC basis) was different as compared to the activity when expressed on a soil mass basis (Figure 5, Figure 6 and Figure 7). The decreased (NR) and increased (PHA, UR) had no clear pattern (GLU), or the changes in their activity were not significant (DHA, CAT). In turn, the enzyme activity, when expressed on s C_ORG_ basis, revealed a similar pattern, as was the case of the activity when expressed on an MBC basis (Figure 5, Figure 6 and Figure 7). 

The specific activity of UR and PHA was the lowest in the Ap, increased with the depth of the soil profiles, and was the highest in the Ck2 horizon. The UR/C_ORG_ ratio was not region specific, while the UR activity, when expressed for MBC, was significantly different in the study regions only in the two the lowest horizons (Figure 6c,d). 

The PHA activity, when expressed on both a C_ORG_ and MBC basis, was significantly higher in the Krajna and Chodzież Lakelands than in the Chełmno region, but only in the Bt2-Ck2 horizons (Figure 7c,d). Conversely, for the UR and PHA activity, the specific NR and β-GLU activity was the highest in the Ap horizons of all of the studied profiles, but decreased in both the Bt1 and Bt2 horizons and then increased once again in the parent material. The study regions only affected the specific NR activity in the Ap horizon; the NR/C_ORG_ ratio was significantly higher in the profiles from Krajna and Chełmno than those in the Chodzież region, while the NR/MBC ratio was significantly higher in the profiles from Krajna than those from the other regions (Figure 6a,b). No clear direction was found in the specific GLU activity as related to the study regions (Figure 7a,b). The specific DHA activity was aligned down the soil profile, and generally, no clear changes were found in the activity as related to the study regions (Figure 5a,b). The specific CAT activity (CAT/C_ORG_ and CAT/MBC) increased across the horizons up to Ck1 and then decreased in the parent material. Similar to the other enzymes (GLU, DHA), the specific CAT activity was not clearly affected in the studied Lakelands (Figure 5c,d). 

#### 3.2.3. Correlation between the Studied Properties

Because the analysis of linear regression that was done to compare the same horizon (e.g., Ap) in the profiles from the same Lakeland and for all of the study regions did not indicate any significant correlation coefficients, we presented and discussed only the analysis that took all of the data into account (all of the horizons down the soil profile), separately for each Lakeland. According to the linear regression analysis, the enzymatic activity was significantly and positively correlated with some of the physicochemical properties (Table 5). The highest positive correlations were calculated between C_ORG_, N_TOT_, MBC, the available K and P content and enzyme activity. A significant but negative relationship was calculated between the soil bulk density and the soil enzyme values, while porosity was correlated positively with all of the enzymes studied.

## 4. Discussion

### 4.1. Physicochemical Properties

The higher pH in the C horizon was likely the result of the presence of carbonates in the glacial till. The bulk density ranged from 1.64 to 1.87 g cm^−3^ (Table 1), which is typical for mineral soil. The bulk density values increased significantly with the depth of the soil profile and reached the highest values in the parent material. The high bulk density indicated considerable soil compaction, which resulted in a very low total porosity in the subsoil horizons. Soil with a porosity value below 40% is considered to be compacted soil. The higher soil density in the subsurface layer, which is caused by mechanical compaction, may be due to the considerable amount of the clay fraction in the Bt horizon [35].

Many authors have pointed out that the depth of the compactness causes changes in the soil profile down to a depth of 60 cm and causes negative interactions between the excessive compactness and potential soil productivity [36,37,38]. 

While the topsoil concentration was obvious for the organic C and total N that is associated with the pool of soil organic matter, the widespread concentration of the limiting mineral nutrients, such as P and K, in the topsoil strongly supports the idea that plants control the distribution of these elements. The relatively high topsoil content of P and K in most of the profiles that were studied support the theory of Jobbágy and Jackson [39] that the most limiting nutrients for plants (those required by plants in high amounts in relation to the soil supply) have the shallowest distributions. The available Mg increased with the depth of the profiles, which suggests that its higher relative contribution is not caused by the plant cycle, but by some abiotic process, such as the preferential retention of leached Mg over Ca by the Al hydroxides in the subsoil [40,41]. The relatively high content of extracted Mg in the Ck horizons could also be connected with the presence of Mg in the carbonate compounds that occurred in the parent material. The content of CaCO_3_ in the C horizons ranged from 7.18 to 11.1% (data not presented). Among the studied available nutrient forms, the shallowest distribution was observed for the available P, which could have been caused by phosphorus fertilization. The low content of available P in the C horizons compared to upper ones was probably an effect of the immobilization of P (as Ca_3_(PO_4_)_2_), which was caused by the high content of calcium ions and by pH values that were higher than 7.0. The vertical distribution of total N was strongly associated with that of the organic carbon. The C:N ratio in the soil profiles tended to decrease with the depth of the soil profile (Table 1), which possibly reflects a greater degree of breakdown and the older age of the humus that is stored in the deeper soil layers than in the surface layers [42]. 

### 4.2. Enzyme Activity in the Soil Profile

In accordance with earlier studies [7,43,44], our results showed a significant decrease in the enzyme activity as a function of soil depth, with the lowest activity generally observed in the C horizons. Although the soil organic carbon content and soil microorganism content and activity are considered to be the major factors that influence the level and distribution of enzymes through a profile, other soil properties such as the available nutrient content, plant cover, soil reaction, soil texture and changes in the soil temperature and moisture significantly affect the enzymatic activity with depth [6,7,8,45]. 

The differences in the enzyme activity among the soil horizons were mainly due to variations in the soil properties, such as the C_ORG_ and N_TOT_ concentration, C/N ratio and microbial biomass content. Soil enzymatic activity decreases with soil depth, mostly due to the fact that the content of C_ORG_ is the highest in surface layers and lower in deeper horizons [46]. Accordingly, decreasing C_ORG_, N_TOT_ and C/N ratios were observed at increased depths for the studied soil profiles. Additionally, the strong positive correlation between soil enzymatic activity and C_ORG_ and N_TOT_ content was supported by an analysis of the correlation (Table 5). The lower soil C/N ratio in the deeper soil horizons indicates that the soil organic matter was more degraded and humified. This fact, together with a lower fresh carbon input by plants into the deeper soil horizons, causes enzymes not to sustain their catalytic capacity in the deeper soil layers compared to that of the surface horizons [8]. The reverse trend in the C/N and MBC/C_ORG_ ratio (Table 1) demonstrated that the last ratio can be used as an index of the C availability for microorganisms, as was proposed earlier [7]. 

It is generally accepted that the soil enzymes are mainly produced by microorganisms, and the MBC has been shown to decrease with increasing soil depth as a result of the decreasing availability of the nutrient concentration in the deeper soil layers [47]. We found a decrease in the MBC content with depth that was closely associated with the decreasing enzymatic activity, and this relationship was confirmed by the high values of the correlation coefficients between all of the enzymes that were studied and the MBC content (Table 5). 

Decreasing of the MBC with depth could be associated with the fact that more of the microbial biomass could have been less active as well as not active or dead due to the extreme deep-soil conditions, such as a higher soil density, lower oxygen concentration and less available carbon and nutrients than in the surface horizons [48]. Based on the literature [48], we can also assume that due to the low competition among microbial communities, the specific microbial taxa are consistently more abundant in deep soils and are preferentially adapted to low-nutrient conditions due to their ability to synthesize and store specific enzymes, such as PHA activity in this study.

The potential enzyme activity (expressed in a soil mass unit) usually decreases with soil depth [2,43,46,49,50], while specific enzyme activity (expressed in organic C or microbial biomass C content) had either similar values throughout the soil profile or an increase in them with depth [10,51]. According to Marinari and Antisari [7], enzyme activity on a soil mass basis estimates the rate at which the product of the enzymatic activity is being made available to microorganisms and plants; as such, it is a quantitative measure. By contrast, the expression of enzymatic activity per C_ORG_ or microbial biomass C unit gives us an estimation of how eligible the organic matter is to be degraded by those enzymes, and that is why it is considered to be an organic matter quality index. We expected that specific enzymatic activity would increase with depth, thereby reflecting greater microbial potential to produce enzymes in response to the decreased availability of carbon and nutrients [52]. As is shown in Figure 5, Figure 6 and Figure 7, soil enzyme activity, when expressed per unit of organic carbon, changed differently with depth compared to the enzyme activity calculated per gram of soil. The relatively high specific enzyme activity per unit of C_ORG_ in the C horizons of the soil profiles studied compared to the upper layers may suggest the presence of substrates and favorable conditions for substrate mineralization (i.e., more degraded and humified soil organic matter) with the contribution of these enzymes. 

We also propose different explanations for the higher level of the PHA and UR activity in the subsurface horizons compared to the surface layers, which is related to the substrate distribution within the soil profile and enzyme stability (binding to different particle size fractions) [8]. First, it may be attributed to the activity of proteins that have leached down from the surface layer and that are associated with the clay minerals. Such bounded soil extracellular enzymes have a high level of stability of their activity, which is generally related to their association with clays; although the activity of clay-immobilized enzymes is generally lower, clay-adsorbed enzymes can retain their catalytic activities even under unfavorable conditions [53,54]. Second, it is possible that the high specific activity of the C- and P-acquiring enzymes in the subsoils reflects constitutive enzyme production [52,55]. Finally, plant C-acquiring enzyme activity could persist in deep soils due to the enhanced mineral stabilization of the enzymes and reduced enzyme turnover rates. From the microbial point of view, the specific enzyme activity, which increases with depth, might indicate that microorganisms may be expending more to acquire less in resource-poor subsoils [2].

The soil enzymatic activity in the soil profiles was significantly influenced by soil density and porosity (Table 5), which was in agreement with other studies [56]. Increased soil density stimulated the DHA and PHA activity, but not the UR activity [56]. Earlier, Marinari et al. [57] reported a significant linear correlation between acid phosphatase and dehydrogenase activity and total soil porosity. As was stated by Pagliai and De Nobili [58], soil enzyme activity is positively influenced by the number of pores that range from 30 to 200 µm. Strong associations between enzymatic activity and soil pores were also found by Kravchenko et al. [59]. In this study, soils were classified as very compacted in the soil profile, which could be the primary character of the parent material [60]. The subsurface horizon of the Luvisols that were investigated, in which the bulk density reached up to 1.87 Mg m^−3^ and the content of the clay fraction ranged from 13.1 to 24.2%, represented soil in a high category of packed density—higher than 1.75 Mg m^−3^ (detailed data not presented). In addition, long-term agricultural soil use usually increases the soil profile compaction compared to the corresponding horizons of natural soil. 

We also considered the relationship between the soil enzymes and cultivated plants, since the roots are a significant source of some enzymes or contribute to favorable conditions for the microbial synthesis of enzymes, e.g., the phosphatase activity in soil [61]. Based on this statement, we can suppose that the behavior of the enzymatic activity throughout the soil profiles (at least in the upper layers) that were studied may be due to the nature of the plant root system. In fact, in our study a statistically significantly higher activity of PHA was found in the profiles with winter rape (deep pile root with numerous but poorly developed lateral roots) compared to winter wheat (shallow, bunched root system), which was also true for the subsoil horizons (Bt1 and Ck1). However, because of the low differences between means (12, 28 and 24% in Ap, Bt1 and Ck1 horizons, respectively), this result has to be treated with caution, since other factors could also contribute. A clear relationship between the PHA activity and root system was found earlier, e.g., the phosphatase activity determined in soils under tea plantations down to a depth of 200 cm did not vary widely since the tea roots extended to that depth [6]. 

We proposed an alternative explanation for higher activity of PHA in the subsurface horizons compared to the surface layers. If soil P availability decreases with depth relative to C and N, as was found in this study, the soil microbes would invest resources in a P-acquiring enzyme, and that is why we observed a relatively high PHA activity in the deeper soil horizons. Further study is, however, required in order to draw clear conclusions regarding the significant level of PHA activity in the deeper soil horizons. 

Due to the wide temperature range found in the study region, soil enzymatic activity, especially in deeper layers of the studied profiles, can be shaped by the activity of psychrophilic enzymes (cold-active enzymes), which are stable and active in the temperature range of 0–30 °C. To function in a changing environment, enzymes have evolved a range of structural features that confer a high level of flexibility compared to thermostable homologs [62]. They need to overcome the reduction of chemical reaction rates induced by low temperatures. This can be reached by increasing the turnover number (*k*_cat_), decreasing substrate affinity (Km) or changing both parameters. The contribution of cold adopted microbes and their enzymes in biological processes occurring in agricultural soils should be considered in the future studies. These kinds of studies seem to be essential for improving our understanding and for modelling the transformation of organic and mineral substances in soil in changeable/cold climates [63].

## 5. Conclusions

The distribution of enzymatic activity with depth indicates that soils demonstrate the most activity in the top 30 cm layer, which has the most favorable content of organic matter and substrates compared to the deeper soil horizons. The enzyme activity in some of the profiles in the study decreased systematically with the soil depth, while in some other profiles, there was a variation between the enzymes in the sharpness of the gradient, and there were no clear changes in the distribution of the enzymatic activity profile. We found that the decreasing availability of carbon and nutrients is likely the principal driver of the decreases in microbial abundance and enzymatic activity with depth. This was evidenced by the strong relationship between soil C, microbial biomass and enzyme activity. The lessivage process significantly affected the soil morphology and clay content but did not influence the enzymatic activity, organic carbon and nutrient concentration (no enrichment was found in the Bt layer). 

Despite the significant differences in some of the studied properties (available P and K, enzymatic activity), no clear direction in their changes was found in the profile horizons of the study regions. 

An investigation of both the physicochemical and biochemical parameters of the profiles should be the subject of further research. The differences in depth distribution among the various enzymes that are related to the substrate distribution within a soil profile and enzyme stability (binding to different particle size fractions) should be investigated. In particular, the distribution of the activity of phosphates should be studied, since this enzyme behaved more diversely than the other enzymes and revealed a high activity even in the parent material. These kinds of studies are essential for improving our understanding and for modeling the transformation of organic and mineral substances as they move down a profile.

## Figures and Tables

**Table 1:** Physicochemical properties down the soil profile (mean for 14 profiles).

**Horizon**	**C_ORG_**	**N_TOT_**	**C/N**	**MBC** **(mg kg^−1^)**	**MBC/C_ORG_**	**Clay** **(%)**	**Bulk Density** **(g cm^−3^)**	**Porosity** **(%)**
	**(g kg^−1^)**							
Ap	9.79 ± 1.06 a*	1.03 ± 0.08 a	9.50 ± 0.54	115.6 ± 38.7 a	1.22 ± 0.44 b	14.1 ± 1.9 c	1.64 ± 0.07 c	35.9 ± 2.2 a
Bt1	2.98 ± 0.66 b	0.34 ±0.08 b	8.76 ± 0.94	38.3 ± 11.6 b	1.35 ± 0.56 b	22.4 ± 2.8 a	1.77 ± 0.05 b	31.3 ± 2.0 ab
Bt2	1.11 ± 0.50 c	0.27 ± 0.07 bc	4.11 ± 1.20	19.4 ± 6.3 c	2.12 ± 1.07 a	19.8 ± 2.0 b	1.77 ± 0.03 b	31.5 ± 1.4 ab
Ck1	0.36 ± 0.09 d	0.10 ± 0.04 c	3.60 ± 0.32	3.92 ± 1.9 d	1.15 ± 0.74 b	15.3 ± 2.4 c	1.85 ± 0.02 a	28.6 ± 0.6 b
Ck2	0.21 ± 0.07 d	0.07 ± 0.02 c	3.00 ± 0.28	2.79 ± 0.7 d	1.47 ± 0.75 b	14.9 ± 1.9 c	1.87 ± 0.01 a	28.3 ± 0.5 b
**Horizon**	**pH in KCl**	**Hh** **[cmol kg^−1^]**	**Basic** **Saturation** **[%]**		**P_AVAIL_**	**K_AVAIL_**	**Mg_AVAIL_**	
					**[mg kg^−1^]**			
Ap	6.65 ± 0.09 b	0.71 ± 0.36 a	95.2 ± 2.9 b		80.4 ± 21.4 a	152.7 ± 84.7 a	92.9 ± 27.2 b	
Bt1	6.38 ± 0.03 b	0.98 ± 0.94 a	94.6 ± 4.9 b		29.9 ± 14.6 b	95.8 ± 30.2 b	120.7 ± 25.7 ab	
Bt2	6.57 ± 0.08 b	0.57 ±0.28 a	96.5 ± 1.6 b		22.8 ± 19.4 b	60.5 ± 14.6 c	127.9 ± 43.0 a	
Ck1	7.21 ± 0.02 a	0.06 ± 0.01 b	99.6 ± 0.3 a		3,9 ± 2.3 c	47.3 ± 16.7 c	105.0 ± 35.5 ab	
Ck2	7.25 ± 0.03 a	0.07 ± 0.01 b	99.7 ± 0.3 a		2.2 ± 1.2 c	49.6 ± 17.4 c	113.6 ± 33.7 ab	

C_ORG_—organic carbon content; N_TOT_—total nitrogen content; MBC—microbial biomass carbon; Hh—hydrolytic acidity; P_AVAIL_—available phosphorus; K_AVAIL_—available potassium; Mg_AVAIL_—available magnesium. *: Different lowercase letters (a, b, c, d) indicate significant differences (*p* < 0.05) between genetic horizons of the profile

**Table 2:** Basic statistics of studied soil oxidoreductases.

Enzyme	Region	Horizon	Mean	Range	SD	CV (%)
Dehydrogenase (mg TPF kg^−1^ h^−1^)	Krajna	Ap	265.1 A^b*	112.9–457.1	129.4	48.1
		Bt1	131.5 Ba	102.4–166.1	26.9	20.4
		Bt2	43.1 Ca	9.9–96.3	34.5	80.0
		Ck1	8.14 Dab	2.0–14.3	5.27	64.7
		Ck2	6.66 Da	4.8–10.9	2.42	36.3
	Chodzież	Ap	238.3 Ab	160.7–313.6	63.6	26.7
		Bt1	123.7 Ba	68.7–176.3	44.0	35.5
		Bt2	44.2 Ca	10.2–73.2	31.5	71.4
		Ck1	9.57 Da	3.0–14.5	5.07	53.0
		Ck2	7.63 Da	3.3–14.0	4.63	60.7
	Dobrzyń	Ap	368.2 Aa	181.6–560.8	154.7	42.0
		Bt1	45.8 Bb	14.6–86.6	29.6	64.6
		Bt2	20.9 Cb	7.2–40.9	14.7	71.0
		Ck1	5.82 Db	3.6–8.4	1.90	32.7
		Ck2	8.30 Da	1.50–11.9	4.66	56.1
Catalase (mg H_2_O_2_ kg^−^^1^ min^−^^1^)	Krajna	Ap	7.02 Aa	4.52–9.52	1.98	28.2
		Bt1	4.12 Ba	3.21–4.82	0.63	15.4
		Bt2	2.43 Ca	1.15–3.25	0.85	34.8
		Ck1	0.75 Dab	0.25–1.10	0.31	41.9
		Ck2	0.18 Dc	0.13–0.22	0.04	21.2
	Chodzież	Ap	6.76 Aa	4.86–8.15	1.53	22.7
		Bt1	3.84 Ba	3.11–4.65	0.76	19.7
		Bt2	2.04 Cab	0.85–3.21	0.96	47.2
		Ck1	0.84 Da	0.53–1.11	0.27	32.9
		Ck2	0.29 Db	0.13–0.42	0.14	47.3
	Dobrzyń	Ap	8.38 Aa	6.05–10.9	1.97	23.47
		Bt1	2.80 Bb	2.11–3.24	0.45	16.1
		Bt2	1.51 Cb	1.02–2.81	0.73	48.7
		Ck1	0.63 Db	0.42–0.86	0.21	32.5
		Ck2	0.41 Da	0.10–0.89	0.36	86.9

Enzymatic activity expressed on a soil mass basis. * Different lowercase letters (a, b, c) indicate significant differences (*p* < 0.05) between the same genetic horizons of profiles in different regions. ^ Different capital letters (A, B, C, D) indicate significant differences (*p* < 0.05) between genetic horizons of the same profile. SD—standard deviation; CV—coefficient of variation; TPF—triphenylformazan

**Table 3:** Basic statistics of N-related enzymatic activity.

Enzyme	Region	Horizon	Mean	Range	SD	CV
Urease (mg N-NH_4_^+^ kg^−^^1^ h^−1^)	Krajna	Ap	2.68 A^b*	1.15–4.59	1.57	58.7
		Bt1	1.06 Ba	0.47–2.69	0.95	89.7
		Bt2	0.34 Cb	0.12–0.52	0.16	48.3
		Ck1	0.36 Cb	0.07–0.99	0.38	106.6
		Ck2	0.26 Cb	0.17–0.35	0.07	26.8
	Chodzież	Ap	3.27 Aa	1.18–5.21	1.77	54.2
		Bt1	1.00 Ba	0.41–1.59	0.56	56.4
		Bt2	0.51 Ca	0.11–1.36	0.58	114.5
		Ck1	0.63 Ca	0.11–0.92	0.36	56.7
		Ck2	0.51 Ca	0.41–0.62	0.09	16.9
	Dobrzyń	Ap	3.76 Aa	1.92–5.94	1.50	39.8
		Bt1	0.52 Bb	0.12–0.77	0.28	54.5
		Bt2	0.27 Bb	0.05–0.40	0.16	58.2
		Ck1	0.25 Bb	0.02–0.51	0.20	80.1
		Ck2	0.36 Bb	0.13–0.67	0.21	57.6
Nitroreductase (mg N-NO_2_ kg^−^^1^ h^−^^1^)	Krajna	Ap	1.98 Aa	0.02–4.07	1.83	92.0
		Bt1	0.09 Ba	0.02–0.21	0.08	90.4
		Bt2	0.02 Ba	0.01–0.04	0.01	23.7
		Ck1	0.03 Ba	0.01–0.08	0.03	101.5
		Ck2	0.01 Ba	0.00–0.03	0.01	83.3
	Chodzież	Ap	0.88 Ab	0.11–2.67	1.22	137.9
		Bt1	0.05 Ab	0.04–0.07	0.01	27.2
		Bt2	0.02 Ba	0.01–0.04	0.01	63.0
		Ck1	0.02 Ba	0.01–0.04	0.01	66.2
		Ck2	0.01 Ba	0.01–0.02	0.01	37.6
	Dobrzyń	Ap	1.59 Aa	0.11–3.10	1.37	86.1
		Bt1	0.02 Bb	0.01–0.04	0.02	68.0
		Bt2	0.02 Ba	0.00–0.05	0.02	89.5
		Ck1	0.02 Ba	0.02–0.03	0.01	24.1
		Ck2	0.01 Ba	0.01–0.02	0.01	55.4

Enzymatic activity expressed on a soil mass basis. * Different lowercase letters (a, b) indicate significant differences (*p* < 0.05) between the same genetic horizons of profiles in different regions. ^ Different capital letters (A, B, C) indicate significant differences (*p* < 0.05) between genetic horizons of the same profile. SD—standard deviation; CV—coefficient of variation.

**Table 4:** Basic statistics of studied soil C- and P-related hydrolases.

Enzyme	Region	Horizon	Mean	Range	SD	CV
Phosphstase (mg *p*NP kg^−^^1^ h^−^^1^)	Krajna	Ap	63.7 A^a*	48.2–91.3	17.6	27.6
		Bt1	31.1 Ba	24.0–44.7	8.01	25.8
		Bt2	25.6 Ba	16.7–36.7	7.19	28.1
		Ck1	26.2 Bb	17.4–38.4	8.48	32.4
		Ck2	25.0 Ba	16.5–37.7	9.93	38.2
	Chodzież	Ap	67.0 Aa	26.6–92.4	28.0	42.5
		Bt1	35.8 Ba	22.3–25.8	13.1	36.5
		Bt2	29.6 Ba	22.9–49.1	13.0	44.1
		Ck1	39.3 Ba	14.4–58.2	18.4	46.8
		Ck2	29.5 Ba	11.3–49.2	17.7	59.9
	Dobrzyń	Ap	64.8 Aa	34.4–88.4	19.8	30.4
		Bt1	34.0 Ba	21.4–41.3	8.36	25.3
		Bt2	24.4 Bc	18.3–33.5	5.75	23.6
		Ck1	17.8 Cb	10.1–23.3	5.23	29.3
		Ck2	14.2 Cb	10.2–18.5	3.22	22.7
β-glucosidase (mg *p*NP kg^−1^ h^−1^)	Krajna	Ap	43.3 Aa	28.4–57.7	11.5	26.6
		Bt1	5.85 Ba	4.31–8.06	1.30	22.2
		Bt2	1.20 Cb	0.40–2.59	0.91	75.6
		Ck1	0.31 Cc	0.08–0.47	0.16	50.8
		Ck2	0.42 Cc	0.16–0.82	0.29	68.0
	Chodzież	Ap	51.0 Aa	31.0–67.0	15.7	30.8
		Bt1	5.18 Ba	2.42–6.55	1.90	36.7
		Bt2	1.80 Ca	0.95–2.69	0.80	44.4
		Ck1	0.75 Cb	0.40–1.28	0.38	48.2
		Ck2	0.78 Cb	0.64–0.86	0.10	12.5
	Dobrzyń	Ap	48.0 Aa	30.2–67.1	14.0	29.1
		Bt1	2.25 Bb	1.77–2.67	0.33	14.5
		Bt2	1.87 Ca	0.69–2.40	0.71	38.1
		Ck1	1.51 Ca	0.51–2.33	0.65	43.2
		Ck2	1.59 Ca	0.82–2.42	0.70	43.8

Enzymatic activity expressed on a soil mass basis. * Different lowercase letters (a, b, c) indicate significant differences (*p* < 0.05) between the same genetic horizons of profiles in different regions. ^ Different capital letters indicate (A, B, C) significant differences (*p* < 0.05) between genetic horizons of the same profile. SD—standard deviation; CV—coefficient of variation; *p*NP—*p*-Nitrophenol.

**Table 5:** Correlation matrix between studied properties (n = 20–25).

	Landlake	C_ORG_	N_TOT_	MBC	pH KCl	Hh	Bulk Density	Porosity	Clay	Available		
										P	K	Mg
DHA	Krajna	0.848 ***	0.825 ***	0.963 ***	−0.500 *	0.435 *	0.815 ***	0.788 ***	-	0.773 ***	0.900 ***	-
	Chodzież	0.914 ***	0.921 ***	0.936 ***	−0.730 **	^-	−0.884 ***	0.870 ***	-	0.774 ***	0.859 ***	-
	Chełmno	0.890 ***	0.869 ***	0.973 ***	−0.450 *	0.400 *	−0.591 **	0.528 **	-	0.730 **	0.681 **	-
CAT	Krajna	0.877 ***	0.904 ***	0.954 ***	−0.636 **	0.609 **	−0.872 ***	0.839 ***	-	0.811 ***	0.905 ***	--
	Chodzież	0.918 ***	0.929 ***	0.942 ***	−0.806 ***	0.502 *	−0.893 ***	0.904 ***	-	0.788 ***	0.846 ***	--
	Chełmno	0.947 ***	0.942 ***	0.973 ***	−0.581 **	0.536 **	−0.721 **	0.647 **	-	0.832 ***	0.738 ***	-
UR	Krajna	0.775 ***	0.768 ***	0.835 ***	-	-	−0.790 ***	0.802 ***	-	0.739 ***	0.841 ***	-
	Chodzież	0.810 ***	0.796 ***	0.704 **	-	-	−0.556 **	0.456 *	-	0.760 ***	0.623 **	−0.459 *
	Chełmno	0.925 ***	0.888***	0.894 ***	−0.483 *	0.407 *	−0.682 **	0.642 **	-	0.736 ***	0.578 **	-
NR	Krajna	0.746 ***	0.698 ***	0.760 ***	-	-	−0.704 **	0.719 **	-	0.692 **	0.696 **	−0.399 *
	Chodzież	0.589 **	0.558 **	-	-	-	-	-	-	0.717 **	0.573 **	−0.483 *
	Chełmno	0.736 ***	0.709 **	0.838 ***	-	-	−0.530 **	0.488 *	-	0.673 **	0.680 **	−0.399 *
GLU	Krajna	0.922 ***	0.906 ***	0.893 ***	-	-	−0.914 ***	0.922 ***	−0.421 *	0.904 ***	0.874 ***	-
	Chodzież	0.931 ***	0.909 ***	0.863 ***	-	-	−0.722 **	0.637 **	−0.502 *	0.889 ***	0.721 **	-
	Chełmno	0.932 ***	0.915 ***	0.760 ***	−0.668**	0.521 **	−0.634 **	0.572 **	-	0.704 **	0.639 **	-
PHA	Krajna	0.825 ***	0.792 ***	0.763 ***	−0.464 *	0.428 *	−0.769 ***	0.762 **	−0.473 *	0.621 **	0.663 **	-
	Chodzież	0.574 **	0.565 *	0.690 **	-	-	−0.669 **	0.675 **	−0.449 *	0.494 *	-	-
	Chełmno	0.906 ***	0.886 ***	0.785 ***	−0.622 **	0.546 **	−0.809 ***	0.753 **	-	0.773 ***	***	-

* *p* < 0.05, ** *p* < 0.01, *** *p* < 0.001; ^—not significant; DHA—dehydrogenase; NR—nitroreductase; CAT—catalase; PHA—phosphatase; GLU—β-glucosidase; UR—urease; C_ORG_—organic carbon; N_TOT_—total nitrogen; MBC—microbial biomass carbon, Hh—hydrolytic acidity; Units of properties are those given under previous tables.

## Data Availability

The data used to support the findings of this study are available from the authors upon request.

## References

[1] Wallenstein M.D., Burns R.G., Dick R.P. (2011). Ecology of extracellular enzyme activities and organic matter degradation in soil: A complex community-driven process. Methods of Soil Enzymology.

[2] Stone M.M., De Forest J.L., Plante A.F. (2014). Changes in extracellular enzyme activity and microbial community structure with soil depth at the Luquillo Critical Zone Observatory. Soil Biol. Biochem..

[3] Baldrian P. (2009). Microbial enzyme-catalyzed processes in soil and their analysis. Plant Soil Environ..

[4] Loeppmann S., Blagodatskaya E., Pausch J. (2016). Enzyme properties down the soil profile—A matter of substrate quality in rhizosphere and detritusphere. Soil Biol. Biochem..

[5] Gianfreda L., Ruggiero P., Nannipieri P., Smalla K. (2006). Enzyme Activities in Soil. Nucleic Acids and Proteins in Soil.

[6] Venkatesan S., Senthurpandian V.K. (2006). Comparison of enzyme activity with depth under tea plantations and forested sites in south India. Geoderma.

[7] Marinari S., Antisari L.V. (2010). Effect of lithological substrate on microbial biomass and enzyme activity in brown soil profiles in the northern Apennines (Italy). Pedobiologia.

[8] Herold N., Schöning I., Berner D., Haslwimmer H., Kandeler E., Michalyik B., Schrumpf M. (2014). Vertical gradient of potential enzymes activities in soil profiles of European beech, Norwaz spruce and Scots pine dominated forest sites. Pedobiologia—J. Soil Ecol..

[9] Senga Y., Hiroki M., Nakamura Y., Watarasi Y., Watanabe Y., Nohara S. (2011). Vertical profiles of DIN, DOC, and microbial activities in the wetland soil of Kushiro Mire, northeastern Japan. Limnology.

[10] Kramer S., Marhan S., Haslwimmer H., Ruess L., Kandeler E. (2013). Temporal variation in surface and subsoil abundance and function of the soil microbial community in an arable soil. Soil Biol. Biochem..

[11] Wang Z., Van Oost K., Govers G. (2015). Predicting the long-term fate of buried organic carbon in colluvial soils. Glob. Biogeochem..

[12] Fontaine S., Barot S., Barré P., Bdioui N., Mary B., Rumpel C. (2007). Stability of organic C in deep layers controlled by fresh C supply. Nature.

[13] Niemi R.M., Vepsäläinen M., Wallenius K., Simpanen S., Alakukku L., Pietola L. (2005). Temporal and soil depth-related variation and soil enzyme activities and in root growth of red clover (*Trifolium pratense*) and timothy (*Phleum pratense*) in the field. Appl. Soil Ecol..

[14] Kabała C., Musztyfaga E. (2015). Clay-illuvial soil in the Polish and international soil classifications. Soil Sci. Ann..

[15] Świtoniak M. (2014). Use of soil profile truncation to estimate influence of accelerated erosion on soil cover transformation in young morainic landscapes, North-Eastern Poland. Catena.

[16] Świtoniak M., Mroczek P., Bednarek R. (2016). Luvisols or Cambisols? Micromorphological study of soil truncation in young morainic landscapes—Case study: Brodnica and Chełmno Lake Districts (North Poland). Catena.

[17] Dreibrodt S., Lomax J., Nelle O., Lubos C., Fischer P., Mitusov A., Reiss S., Radtke U., Nadeau M., Grootes P.M. (2010). Are mid-latitude slopes sensitive to climatic oscillations? Implications from an Early Holocene sequence of slope deposits and buried soils from eastern Germany. Geomorphology.

[18] Kittel P. (2014). Slope deposits as an indicator of anthropopressure in the light of research in Central Poland. Quat. Int..

[19] IUSS Working Group WRB (2015). World References Base for Soil Resources.

[20] Kobierski M. (2013). Morphology, Properties and Mineralogical Composition of Eroded Luvisols in Selected Morainic Areas of the Kujavian and Pomeranian Province. Habilitation Thesis.

[21] Polish Norm PN-ISO 11277 (2005). Soil Quality—Determination of Particle Size Distribution in Mineral Soil Material—Method by Sieving and Sedimentation.

[22] Polish Norm PN-ISO 10390 (1997). Soil Quality—Determination of Soil pH.

[23] Burt R. (2004). Soil Survey Laboratory Methods Manual.

[24] Egnér H., Riehm H., Domingo W.R. (1960). Studies concerning the chemical analysis of soils as background for soil nutrient assessment II: Chemical extracting methods to determinate the phosphorous and potassium content of soil. Kungl. Lantbr. Ann..

[25] Hao X., Ball B.C., Culley J.L.B., Carter M.R., Parkin G.W., Carter M.R., Gregorich E.G. (2008). Soil Density and Porosity. Soil Sampling and Methods of Analysis.

[26] Thalmann A. (1968). Zur Methodik der Bestimmung der Dehydrodgenaseaktivität im Boden mittels Triphenyltetrazoliumchlorid (TTC). Landwirtsch. Forsch..

[27] Kandeler E., Scinner F., Öhlinger R., Kandeler E., Margesin R. (1995). Enzymes Involved in Nitrogen Metabolism. Methods in Soil Biology.

[28] Johnson J.L., Temple K.L. (1964). Some variables affecting measurement of catalase activity in soil. Soil Sci. Soc. Am. Proc..

[29] Tabatabai M.A., Bremner J.M. (1969). Use of p-nitrophenylophosphate for assay of soil phosphatase activity. Soil Biol. Biochem..

[30] Eivazi F., Tabatabai M.A. (1988). Glucosidases and galactosidases in soils. Soil Biol. Biochem..

[31] Kandeler E., Gerber H. (1988). Short-term assay of soil urease activity using colorimetric determination of ammonia. Biol. Fertil. Soils.

[32] Vance E.D., Brookes P.C., Jenkinsen D.S. (1987). An extraction method for measuring soil microbial biomass C. Soil Biol. Biochem..

[33] Anderson T.H., Domsch K.H. (1989). Ratios of microbial biomass carbon to total organic carbon in arable soils. Soil Biol. Biochem..

[34] Wilding L.P., Nielsen D.R., Bouma J. (1985). Spatial variability: Its documentation, accommodation, and implication to soil surveys. Soil Spatial Variability.

[35] Hamza M.A., Anderson W.K. (2005). Soil compaction in cropping systems: A review of the nature, causes and possible solutions. Soil Till. Res..

[36] Flowers M., Lal R. (1998). Axle load and tillage effect on soil physical properties and soybean grain yield on a mollic ochraqualf in Northwest Ohio. Soil Till. Res..

[37] Gregorich E.G., Lapen D.R., Ma B.L., McLaughlin N.B., VandenBygaart A.J. (2011). Soil and crop response to varying levels of compaction, nitrogen fertilization, and clay content. Soil Sci. Soc. Am. J..

[38] Lipiec J. Crop responses to soil compaction. Proceedings of the NJF Seminar 448 on Soil Compaction—Effects on Soil Functions and Strategies for Prevention.

[39] Jobbágy E., Jackson R.B. (2001). The distribution of soil nutrient with depth: Global patterns and the imprint of plants. Biogeochemistry.

[40] Smeck N.E., Sajf H.T., Bigham J.M. (1994). Formation of a transient magnesium-aluminium double hydroxide in soils of southeastern Ohio. Soil Sci. Soc. Am. J..

[41] Sajf H.T., Smeck N.E., Bigham J.M. (1997). Pedogenic influence on base saturation and calcium/magnesium ratios in soils of southeastern Ohio. Soil Sci. Soc. Am. J..

[42] Callesen I., Raulund-Rasmussen K., Westman C.J., Tau-Strand L. (2007). Nitrogen pools and C:N ratios in well-drained Nordic forest soils related to climate and soil texture. Environ. Res..

[43] Kizilkaya R., Dengiz O. (2010). Variation of land use and land cover effects on some soil physic-chemical characteristics and soil enzyme activity. Zemdirbystre-Agriculture.

[44] Steinweg J.M., Dukes J.S., Paul E.A., Wallenstein M.D. (2013). Microbial responses to multi-factor climate change: Effects on soil enzymes. Front. Microbiol..

[45] Goberna M., Sánchez J., Pascual J.A., García C. (2006). Surface and subsurface organic carbon, microbial biomass and activity in a forest soil sequence. Soil Biol. Biochem..

[46] Taylor J.P., Wilson M.S., Mills M.S., Burns R.G. (2002). Comparison of microbial numbers and enzymatic activities in surface soils and subsoils using various techniques. Soil Biol. Biochem..

[47] Ge C.R., Xue D., Yao H.Y. (2010). Microbial biomass, community diversity, and enzyme activities in response to urea application in tea orchard soils. Commun. Soil Sci. Plant Anal..

[48] Brewer T.E., Aronson E.L., Arogyaswamy K., Billings S.A., Botthoff J.K., Campbell A.N., Dove N.C., Fairbanks D., Gallery R.E., Hart S.C. (2019). Ecological and genomic attributes of novel bacterial taxa that thrive in subsurface soil horizons. mBio.

[49] Lemanowicz J. (2018). Dynamics of phosphorus content and the activity of phosphatase in forest soil in the sustained nitrogen compounds emissions zone. Environ. Sci. Pollut. Res..

[50] Lemanowicz J., Siwik-Ziomek A., Koper J. (2019). Enzymatic variation of soils exposed to the impact of the soda plant in terms of biochemical parameters. Int. J. Environ. Sci. Technol..

[51] Gelsomino A., Azzellino A. (2011). Multivariate analysis of soils: Microbial biomass, metabolic activity, and bacterial-community structure and their relationships with soil depth and type. J. Plant Nutr. Soil Sci..

[52] Allison S.D., Weintraub M.N., Gartner T.B., Waldrop M.P., Shukla G.C., Varma A. (2011). Evolutionary economic principles as regulators of soil enzyme production and ecosystem function. Soil Enzymology.

[53] Allison S.D. (2006). Soil minerals and humic acids alter enzyme stability: Implications for ecosystem processes. Biogeochemistry.

[54] Gianfreda L., Rao M., Dick R.P. (2011). Stabilizing enzymes as synthetic complexes. Methods of Soil Enzymology.

[55] Sinsabaugh R.L., Shah J.J.F. (2012). Ecoenzymatic stoichiometry and ecological theory. Annu. Rev. Ecol. Evol. Syst..

[56] Pupin B., da Silva Freddi O., Nahas E. (2009). Microbial alteration of the soil influenced by induced compaction. Rev. Bras. Ciênc. Solo.

[57] Marinari S., Masciandaro G., Ceccanti B., Grego S. (2000). Influence of organic and mineral fertilizers on soil biological and physical properties. Bioresour. Technol..

[58] Pagliai M., De Nobili M. (1993). Relationship between soil porosity, root development and soil enzyme activity in cultivated soils. Geoderma.

[59] Kravchenko A.N., Guber A.K., Razavi B.S., Koestel J., Quigley M.Y., Robertson G.P. (2019). Microbial spatial footprint as a driver of soil carbon stabilization. Nat. Commun..

[60] Kobierski M., Wojtasik M. (2009). Organic and inorganic carbon densities in arable and orchard soils in selected mesoregions of the South-Baltic Lakeland. Soil Sci. Ann..

[61] Egamberdieva D., Renella G., Wirth S., Islam R., Shukla G., Varma A. (2011). Enzyme activities in the Rhizosphere of Plants. Soil Enzymology.

[62] Zanphorlin L.M., de Giuseppe P.O., Honorato R.V., Costa Tonoli C.C., Fattori J., Crespin E., Lopes de Oliveira P.S., Ruller R., Murakami M.T. (2016). Oligomerization as a strategy for cold adaptation: Structure and dynamics of the GH1 β-glucosidase from *Exiguobacterium antarcticum* B7. Sci. Rep..

[63] Siddiqui K.S., Cavicchioli R. (2006). Cold-adopted enzymes. Annu. Rev. Biochem..

